# Prosocial Behavior in Preschoolers: Effects of Early Socialization Experiences With Peers

**DOI:** 10.3389/fpsyg.2022.840080

**Published:** 2022-02-17

**Authors:** Nicoletta Salerni, Claudia Caprin

**Affiliations:** Department of Psychology, University of Milano-Bicocca, Milan, Italy

**Keywords:** prosocial behavior, daycare attendance, social-emotional competence, behavioral skills, preschool children

## Abstract

Over the last decades, many studies had focused on the psychological outcomes of children who have received early socialization outside of the family context, highlighting that the daycare experience can both positively and negatively influence the child’s social-emotional development. Despite the number of studies conducted, there is a lack of observational research on this topic. The purpose of this study is to investigate whether the early daycare experience can influence the prosocial behaviors that children exhibit during free-play social interactions with peers, focusing on their quantity and quality. In addition, the associations between the enactment of prosocial behaviors and social-emotional and behavioral competence were investigated. 160 preschoolers, 77 of whom had previously attended daycare, participated in the study and were focally observed during two free play sessions with peers. Each prosocial behavior was identified and subsequently classified using a coding scheme designed to consider the self-initiated or required origin of prosocial actions and their underlying motive. Emotion comprehension was measured using a standardized test, while social-emotional and behavioral competence was assessed using a questionnaire filled out by teachers. The main findings showed that children who had attended daycare had higher anger and aggression scores than those who had not, who, in turn, were rated by their teachers as having more internalizing behaviors. These characteristics seemed to account for the differences found in the tendency to act prosocial acts in response to a peer’s request, which was lower in children who had a previous daycare experience. Moreover, early socialization outside of the family context appeared to foster the comprehension of others’ intent to achieve emotional or instrumental personal goals and, at the same time, to reduce conventional/affiliative prosocial acts. Overall, this study suggested that the incidental effects of daycare on prosocial behavior might be canceled due to the peculiar social-emotional and behavioral characteristics of the two groups of children.

## Introduction

Prosociality refers to a broad class of positive social behaviors that have been deeply investigated in the last 50 years. Currently, this term is used to label intentional acts that share the intent to benefit another person to satisfy his/her needs (Eisenberg et al., 2006; Tomasello, 2009, 2016, 2019; Svetlova et al., 2010; Dunfield and Kuhlmeier, 2013). In this sense, the adjectives prosocial and altruistic are often used in the literature as synonyms even though they subsume only partially overlapping meanings: in fact, altruism implies a personal cost for the actor and, consequently, altruistic behaviors should be considered a subcategory of prosocial ones (Eisenberg et al., 2006).

As suggested by Dunfield and Kuhlmeier (2013) engaging in prosocial behaviors can be considered a three-step process: (1) recognizing the presence of cues (behavioral or situational) that suggest another individual is experiencing a negative state of need, desire, or distress; (2) identifying the appropriate intervention to help the other achieve alleviation of his/her negative state; and (3) being motivated to engage in prosocial behaviors on his/her favor.

Contrary to the common view that children only gradually learn to be prosocially responsive to others as a function of successful socialization, the early emergence of a wide range of spontaneous positive behaviors is well documented by the end of the first year of life. In fact, concerning the first step of engaging in prosocial behavior, studies have shown that despite poor social cognition skills and the developing ability to differentiate one’s own internal states from those of others, a child’s attitude toward prosocial behavior is so strong that simple cues to affiliation can elicit both helping and sharing behaviors and deliberate efforts to comfort others in need. Indeed, from the beginning of the second year, toddlers can spontaneously provide both useful information, through the pointing gesture, and instrumental help without explicit requests or expectation of rewards (Svetlova et al., 2010; Newton et al., 2014). In other words, children recognize the need for help in others early on, even though the forms of response show different developmental patterns from infancy to preschool age and little cross-task correlation (Dunfield and Kuhlmeier, 2010, 2013).

Regarding the second step in the process that leads to the production of prosocial behaviors, there are many different actions that individuals can engage in to benefit others so that different classifications can be found in the literature. About this, Tomasello (2009) has suggested that prosocial behavior can be classified by distinguishing three main categories of helping others, namely, with services, goods, and information. Dunfield and Kuhlmeier (2013) also identified three main classes of human needs: instrumental need (being unable to complete a goal-directed behavior if alone), emotional distress regulation (experiencing an unpleasant emotional state), and material desire (being unable to acquire the desired resource). Despite this, most studies have focused on one or two specific forms of behavior (among which helping, comforting, sharing material resources, and providing information are those most investigated), then generalizing the results obtained to the broader class of prosocial behaviors. In addition, it is important to note that previous studies which have examined and compared multiple forms of prosocial acts have reported mixed results and only a few of them found interrelationships between the different behaviors considered, however low. For this reason, the literature often reports discrepant interpretations regarding the developmental trajectories of prosocial behavior (Brownell et al., 2013; Dunfield and Kuhlmeier, 2013; Flook et al., 2019) from early infancy to middle childhood so that, despite the large body of work that has been conducted on this topic, our general understanding of this social phenomenon is still not very straightforward.

Concerning the underlying motivation for engaging in prosocial behaviors, recent studies, primarily focused on the first 5 years of life, have shown that, during development, the enactment of these behaviors is, in a sense, “normed” by social rules and by the understanding of which is the most appropriate behavior to exhibit in specific circumstances; moreover, the expectation that the same behavior will be directed, in turn, toward oneself becomes important (reciprocity social rule). With development, therefore, there should be a general increase in the enactment of prosocial behaviors, but also a greater selectivity with respect to the recipient (Tomasello, 2009, 2016, 2019; Kuhlmeier et al., 2014).

On this point, the recent debate is oriented toward some specific issues: one of the main questions concerns the relationship between prosociality and social competence; moreover, another debated matter relates to the effect of social norms on the natural tendency to be responsive to the needs and/or desires of others, enacting behaviors in their favor (Laible et al., 2014; Newton et al., 2014; Tomasello, 2016). Previous studies have found positive links between prosocial behavior and social competence from preschool to late elementary school: prosociality serves to create cohesion among people, that is the goal of social competence (Laible et al., 2014; Flook et al., 2019), and findings have shown that prosocial children have more frequent positive interactions with peers and obtain high social preference (Chung-Hall and Chen, 2010; Farina and Belacchi, 2021); prosocial preschool children also show increased development of some abilities considered to be relevant to social competence, such as communicative-linguistic skills (Conte et al., 2018; Bouchard et al., 2020) and those concerning Theory of Mind and emotion understanding (Imuta et al., 2016; Conte et al., 2018; Traverso et al., 2020). However, from a developmental perspective, it should be assumed that the characteristics that define social competence in one period might be different from those that characterize other developmental phases.

In light of this, the preschool period can be considered particularly interesting to investigate these issues since many maturational and experiential changes occur rapidly in this developmental phase, allowing quantitative and qualitative “jumps” in many social-emotional skills, including emotion comprehension and emotional and behavioral self-regulation (Hartup, 2011; Rose-Krasnor and Denham, 2011).

Moreover, considering this period of life it is also possible to investigate whether and how early socialization may foster or inhibit prosociality (Tomasello, 2016, 2019; Flook et al., 2019; Schmerse and Hepach, 2021). Many studies have focused on the psychological outcomes that may emerge if children also receive early socialization outside of the family context, i.e., in daycare, where they have various experiences with the peer group, under the guidance of one or more teachers (McCormick, 2018; Bleiker et al., 2019). This environment is structured according to specific guidelines that are geared toward good practices to promote the overall wellbeing and development of children, even though attending daycare means, for the child, experiencing daily separation from significant family members and dealing with the resulting stress. In addition, the daily experiences within it, especially those of free play with peers, can represent pleasant and enriching opportunities for cooperation but, at the same time, also for conflict, depending on the circumstances (Hartup, 2011).

In this regard, available evidence testifies that the daycare experience can either positively or negatively influence child development, or not influence it at all, as shown by various studies concerning cognitive and communicative-linguistic development (Bulgarelli and Molina, 2016; Bleiker et al., 2019). With specific reference to social-emotional and behavioral skills, the main hypothesis is that early relational experience with an extended peer group can foster general capacities for understanding other children and their needs and, consequently, promote prosocial behavior. In line with this hypothesis, a few studies have shown that the daycare experience promotes the comprehension of both others’ state of mind and emotions (Rose-Krasnor and Denham, 2011).

It is important to underline that also daycare teachers often promote prosocial behavior among children (Quigley and Hall, 2016; McCormick, 2018; Bleiker et al., 2019; Schmerse and Hepach, 2021), focusing their attention on others’ distress as the mothers usually did (Döge and Keller, 2014) and encouraging helping behaviors (Grazzani et al., 2016) more than cooperation during structured activities or play (Li et al., 2016).

However, studies in this area of investigation are quite scarce. A recent study by Schmerse and Hepach (2021) found that both parent and teacher socialization goals, but also the social climate among peers in the classroom group, predicts young children’s concern for others and their subsequent acts of help between the ages of two and four: children who received good quality of care in both the family and daycare environments and who experienced a pleasant climate of peer interaction exhibited more prosocial behavior than children who grew up early only in the family context. These findings are consistent with those from previous studies that showed good daycare experience can promote prosociality (Hyson and Taylor, 2011; Grazzani et al., 2016; Quigley and Hall, 2016; McCormick, 2018). In contrast, Bleiker et al. (2019) highlighted that prosocial behavior of children aged 18-24 months who attended daycare did not differ from that of family-raised peers; furthermore, a study by Pingault et al. (2015) showed that, at age six, children with daycare experience were more sociable but equally prosocial than others.

At the same time, it has also been well established that children who attend daycare are prone to be more aggressive and disobedient than those who grow up in families (NICHD Early Child Care Research Network, 2002, 2003; Varin, 2007; McCartney et al., 2010). It is hypothesized that this increase in aggression depends primarily on the number of hours spent in that educational context (Ansari, 2018), as evidenced by the direct association found between the amount of daily time spent in the daycare center and children’s cortisol levels (Watamura et al., 2003). The occurrence of such aggressive behaviors can certainly be an indicator of discomfort and stress due to the long period spent away from the family; however, other reasons can also be at the origin of such behaviors since the child also feels the desire to assert him/herself and his/her independence from others. According to evolutionary theory, Hawley and Vaughn (2003) argued that humans are often in a competitive state for resources; therefore, aggressive behavior and trait aggression could be interpreted as adaptive in the sense of promoting access to physical and psychological resources in preschoolers who are not yet able to negotiate with others. Furthermore, social competence and social dominance are closely intertwined during childhood, and aggressive behavior may be characteristic of socially competent preschoolers. In other words, prosocial and aggressive behaviors can be viewed as two different ways of interacting with peers that children can use to achieve their goals and satisfy their needs, depending on the circumstances: one more cooperative and one more coercive (Ostrov and Crick, 2007).

To date, it is unclear how long these effects of daycare may influence subsequent behavior, i.e., whether the experience of daycare can be considered a protective or risk factor regarding different social skills (Filho et al., 2016; Muñoz et al., 2017; Ansari, 2018). In addition, it is important to note that most research has been conducted through questionnaires completed by teachers and parents, and there is a lack of observational research on the prosocial behaviors that children exhibit every day during free-play social interactions with their peers (Conte et al., 2018; Bouchard et al., 2020).

### Aims of the Study

Moving from the above considerations, the present study was designed to achieve two primary goals. The first was to investigate the possible influence of the daycare experience on preschoolers’ social-emotional skills, looking at the long-term effects of this experience by comparing children who had attended daycare in early childhood with those who had been experiencing family care. The focus was on the prosocial behaviors displayed by children during interactions with peers and, more specifically, the attention has been paid not only to the productivity associated with such positive social behaviors but also to their quality, considering both their spontaneous or on-request nature and the various underlying motives. Since this type of investigation has usually adopted indirect methods of assessment, using direct observation of children’s behaviors might help disambiguate some of the mixed results reported in the literature, by overcoming some of the limitations associated with this type of measurement.

In addition, to better understand the influence that daycare attendance might have on prosociality, the differences in certain social-emotional and behavioral skills of children who had such early socialization experience and those who had not were also examined. At the same time, the synchronic interrelations between prosocial behavior and social-emotional and behavioral competencies were investigated. Several studies highlighted that negative emotionality could inhibit prosocial behaviors and that children who have attended daycare show higher levels of aggression and disobedience. Therefore, it is reasonable to hypothesize that these aspects might have a different impact on the prosocial behavior observed in these two groups of children.

## Materials and Methods

### Participants

The study involved 160 children (Males = 83) aged between 3 and 6 years (*M* = 4.74; *SD* = 0.86) recruited from eight kindergartens in four cities in northern Italy.

The research project has been presented to both school administrators and teachers and following their approval the informed consent documents were handed out to the parents of each child. In addition, they were given a socio-demographic questionnaire to obtain information regarding the age of the children, their previous attendance or non-attendance at the daycare, the presence or absence of siblings, and both the educational level and occupation of the parents.

The socioeconomic status of the families was assessed based on maternal and paternal employment and educational level of both parents: 29.4% were low-SES families (parents with compulsory education, manual occupation, or low responsibility job), 56.9% were mid-SES (parents with a high school diploma and middle management profession), and the remaining 13.8% were high-SES (parents with a bachelor’s degree, or higher, and a profession of high responsibility). In cases of discrepancy between parental status, priority was given to maternal characteristics. Most of the participants had siblings (78.8%).

Participants were divided into two groups based on whether or not they had previously attended daycare: the first group (G1: Males = 36; Females = 41) included children with early group experience, whose ages ranged from 3.14 to 6.13 years (*M* = 4.62; *SD* = 0.89), while the second group (G2: Males = 47; Females = 36) consisted of children with no early group experience, aged between 3.13 and 6.26 years (*M* = 4.87; *SD* = 0.82). Children in the two groups did not significantly differ for age (*t*_(162)_ = −1.872; *p* = 0.063) and gender distribution ( $\chi_{\left(1\right)}^{2}$ = 1.560, *p* = 0.212).

The study met ethical guidelines for human subject protections, including adherence to the legal requirements of Italy, and received formal approval by the local Research Ethical Committee of the University of Milano-Bicocca.

### Procedure and Instruments

The research was conducted using different data collection methods, both direct and indirect. Specifically, direct observation was used to examine prosocial behaviors, while social-emotional and behavioral skills were assessed *via* an indirect observation instrument. Finally, emotion understanding was measured using a standardized test.

#### Naturalistic Observations and Coding System of Prosocial Behaviors

Before beginning with the observational sessions, the observers were adequately trained, for approximately 2 months, by one of the authors using a series of videos of peer interactions. The training ended when they reached a 90% agreement rate (compared to criterion protocols) regarding both the identification and the classification of prosocial behaviors. Subsequently, in pairs, they carried out a period of familiarization, spending a minimum of 3 days in the classroom to reduce the children’s reactivity to their presence. Each child was focally observed in two sessions, each lasting 20 min, during free play activities with peers that can be considered qualitatively and structurally similar across the different kindergartens involved in the study. To collect a representative sample of the child’s behaviors, each observational session was conducted at about 15-day intervals, one scheduled in the morning and one in the afternoon. Each observation started at the time the target child began to interact freely with peers and was suspended if he or she moved away from other children or engaged in solitary play; so, the time that the focal child did not spend in social exchanges was not considered in determining the temporal duration of the observation.

Each observer described all social behaviors exhibited by the target child using an audio recorder, also including the antecedents of the behaviors themselves and the reactions of all children involved in the interaction. On approximately the same day the observations were conducted, the observers transcribed their audio descriptions and drew up the narrative observational protocols, adding more details as possible and paying particular attention to communicative intentions and other fundamental aspects of non-verbal communication.

From the transcripts, all prosocial behaviors produced by each child, defined as voluntary actions intended to benefit another person by improving their wellbeing and reducing their state of distress, were identified firstly. These behaviors were, then, classified using a coding system specifically developed by the authors for the purpose of the study, considering both the self-initiated or required origins of prosocial actions and their underlying motives.

More specifically, three main motives were identified considering other’s needs:

- affiliative or conventional: concerns cases in which the action is predominantly driven by social rules, that is the prosocial behavior is more closely associated with socialization than with the others’ wellbeing (i.e., greeting and hugging a classmate who is coming up);
- empathic: refers to actions prompted by a peer’s emotional distress and need for hetero regulation (i.e., comforting a peer after a physical accident, or taking the role of peacemaker in other children’s conflicts)
- other’s desire: pertains to behaviors enacted as a result of understanding the other’s intent to achieve instrumental and/or cognitive goals (i.e., retrieving an object that another child is looking for and giving it to him/her; providing information).

Finally, positive social actions omitted by children (both those that followed an explicit request and those whose antecedent was an implicit request) were considered.

From this classification, the following measures were then calculated: (1) the total frequency of prosocial behaviors; (2) the proportion of self-initiated prosocial behaviors out of the total number of prosocial behaviors produced; (3) the proportion of required prosocial behaviors out of the total number of requests received (both explicit and implicit); and (4) the proportions of prosocial behaviors associated with each motive category out of the total number of prosocial behaviors enacted.

Interrater reliability was calculated using the percentage of agreement on the occurrence of prosocial behaviors (87.24%) and Cohen’s *κ* coefficient on their classification (*κ* = 0, 89).

#### Social-Emotional and Behavioral Competence

In order to assess children’s social-emotional and behavioral skills, teachers were asked to fill in the Italian version of the Social Competence and Behavior Evaluation-Short Form questionnaire (SCBE-30; LaFreniere and Dumas, 1996; Sette et al., 2015), designed to assess social competence, emotion regulation and expression, as well as adjustment difficulties in children between 30 and 78 months of age. The scale is composed of 30 items referable to three subscales, each of which includes 10 items; in particular, the subscales Anxiety-Withdrawal and Anger-Aggression investigate maladaptive behavior patterns, while the Social Competence subscale explores adaptive behaviors.

For each item, teachers were asked to indicate the frequency a child exhibited a target behavior or emotional state on a 6-point Likert scale ranging from 1 (“never”) to 6 (“always”). Scores for each subscale were considered for analysis.

#### Emotion Comprehension

Each child’s understanding of emotion was assessed by means of the Test of Emotion Comprehension, Italian version (TEC; Pons and Harris, 2000; Albanese and Molina, 2008), which is appropriate for children ages 3–11 years. It refers to nine components regarding the nature of emotions (i.e., recognition of basic emotions and understanding of mixed emotions), the causes of emotions (i.e., external causes, memories, desires, beliefs, and moral values), and the ability to control the expression of emotions (i.e., regulation of an experienced emotion and discrepancy between felt and expressed emotions).

The test consists of a picture book composed of a series of cartoon scenarios, available in both male and female versions, shown at the top of each page; at the bottom, four possible emotional outcomes represented by as many facial expressions (*“happy,” “sad,” “angry,”* and *“scared”*) are placed.

The child is read a short story while looking at the scenario of the cartoon and, afterward, is asked to point out the facial expression that corresponds to the emotion felt by the character in the story. One point is assigned for each component answered correctly.

Children were individually tested in a separate room at their kindergartens and each assessment typically lasted about 15 min. Overall, data collection was conducted by two researchers who were specifically trained to ensure both consistency and uniformity in the administration of the test and to transcript and code children’s responses according to the scoring system.

Analyses were carried out using the global score (which can range from 0 to 9) obtained by summing the sub-scores for each component.

## Results

### Statistical Analyses

IMB SPSS Statistic 27 was used to conduct data analyses. Although the gender variable was equidistributed within the two groups of children examined, a series of preliminary *t*-tests were conducted to assess its possible influence on all the observed variables. None of the differences attributable to the gender of the participants were found to be statistically significant. Moreover, given the large variability associated with participants’ age, correlational analyses were also preliminarily conducted to test its association with the measures considered; from the results obtained, this variable was then controlled in all the analyses performed.

A multivariate analysis of covariance was carried out to assess the first aim regarding the presence of differences in the quantity and quality of prosocial acts as a function of daycare attendance. A similar analysis was conducted to investigate any differences in social-emotional and behavioral competencies in the two groups of children.

Finally, partial correlations have been performed to explore the concurrent associations among prosocial behavior and social-emotional and behavioral competencies.

### Prosocial Behavior

The first group of analyses focused on the productivity of prosocial behaviors, both in their totality and with respect to their spontaneous or required origin, to verify the presence of any differences between children who attended daycare centers and those who did not.

Since the frequency of prosocial behaviors observed during social exchanges positively correlated with age (*r* = 0.230, *p* = 0.003), a multivariate analysis of covariance was conducted with daycare attendance as the independent variable, the total number of prosocial acts, and the proportion of both self-initiated and required prosocial behaviors as dependent variables, and age in months as the covariate.

There were no statistically significant differences between the two groups of children in either the total number of prosocial behaviors produced [*F*_(1)_ = 0.621, *p* = 0.432, *η*^2^ = 0.004] or the proportion of those enacted spontaneously [*F*_(1)_ = 1.227, *p* = 0.270, *η*^2^ = 0.008]. In contrast, children who had attended daycare were significantly less likely to engage in prosocial behaviors in response to implicit and explicit requests from other children [*F*_(1)_ = 6.29, *p* = 0.011, *η*^2^ = 0.043].

A similar analysis was conducted to verify the effect, if any, of daycare attendance on the specific motivations underlying the enactment of children’s prosocial behaviors. Results showed that children who had previously attended daycare produced a higher proportion of prosocial behaviors driven by the fulfillment of a desire expressed by another individual, compared to those that had not such socialization experience [*F*_(1)_ = 5.735, *p* = 0.018, *η*^2^ = 0.035]. Moreover, the same children showed prosocial behaviors generated by affiliative/conventional motivations in smaller proportions, although this difference is only marginally statistically significant [*F*_(1)_ = 3.010, *p* = 0.085, *η*^2^ = 0.019]. No statistically significant difference was found in prosocial behaviors associated with empathic motives [*F*_(1)_ = 1.156, *p* = 0.284, *η*^2^ = 0.007].

The descriptive statistics of all the measures considered were summarized in Table 1.

**Table 1:** Descriptive data for prosocial behavior measures.

	All participants (*n* = 160)			G1 (daycare attendance; *n* = 77)			G2 (no daycare attendance; *n* = 83)		
	*M*	SD	Range	*M*	SD	Range	*M*	SD	Range
Total prosocial behaviors	18.19	9.82	1–46	17.12	10.68	1–45	19.19	8.89	3–46
Spontaneous prosocial behaviors	0.78	0.16	0.00–1.00	0.77	0.18	0.00–1.00	0.80	0.14	0.31–1.00
Requested prosocial behaviors^*^	0.68	0.31	0.00–1.00	0.61	0.31	0.00–1.00	0.74	0.30	0.00–1.00
Affiliative/conventional motive	0.50	0.20	0.00–1.00	0.48	0.21	0.00–1.00	0.53	0.18	0.00–0.85
Empathic motive	0.07	0.11	0.00–0.67	0.06	0.09	0.00–0.54	0.08	0.13	0.00–0.67
Other’s desire	0.43	0.20	0.00–1.00	0.46	0.22	0.00–1.00	0.39	0.16	0.00–0.77

* *n* *was slightly different as some children did not receive implicit or explicit requests from their peers. The related values were: *n* = 152 (all participants); *n* = 73 (G1); *n* = 79 (G2)*.

### Social-Emotional and Behavioral Competence

An additional set of analyses was conducted to investigate whether daycare attendance contributed to influencing children’s social-emotional and behavioral competence. Table 2 summarizes the descriptive statistics of all measures considered.

**Table 2:** Descriptive data for social-emotional and behavioral measures.

	All participants (*n* = 160)			G1 (daycare attendance; *n* = 77)			G2 (no daycare attendance; *n* = 83)		
	*M*	SD	Range	*M*	SD	Range	*M*	SD	Range
Anxiety-withdrawal score	20.28	6.71	10–46	19.40	5.75	10–33	21.10	7.44	10–46
Anger-aggression score	18.51	8.31	10–52	20.66	9.55	10–52	16.52	6.39	10–41
Social competence score	36.79	10.68	15–59	37.19	10.83	15–59	36.41	10.58	17–57
TEC score	4.71	2.06	0–9	4.70	2.05	0–9	4.71	2.09	0–9

A series of preliminary correlational analyses were performed to test for the relationships between the three components of social-emotional and behavioral competencies assessed using the SCBE-30 and age in months.

The results showed that the scores children obtained on the Social Competence subscale significantly increased as a function of age (*r* = 0.386, *p* < 0.001), whereas an opposite pattern of association was found regarding the Anxiety-Withdrawal component (*r* = −0.219, *p* = 0.005). The correlation calculated between the Anger-Aggression score and children’s age was not statistically significant (*r* = −0.077, *p* = 0.336).

Given these results, a multivariate analysis of covariance was performed considering the scores obtained in each of the subscales of the SCBE-30 as the dependent variables, daycare attendance as the independent one, and age as the covariate. Children who had attended daycare were characterized by higher scores on the Anger-Aggression subscale than their peers who had not this socialization experience [*F*_(1)_ = 9.747, *p* = 0.002, *η*^2^ = 0.058], whose, in turn, displayed higher scores concerning the Anxiety-Withdrawal component [*F*_(1)_ = 4.459, *p* = 0.036, *η*^2^ = 0.028]. No statistically significant difference was found between the two groups concerning the Social Competence score [*F*_(1)_ = 1.777, *p* = 0.184, *η*^2^ = 0.011].

In addition, about emotional competence, a univariate analysis of covariance was carried out considering the TEC score as the dependent variable, the daycare attendance as the independent factor, and age in months as the covariate since this last variable resulted positively correlated with the emotional competence measure (*r* = 0.500, *p* < 0.001). The results obtained did not show a statistically significant main effect of the independent variable on children’s emotional comprehension [*F*_(1)_ = 1.208, *p* = 0.273, *η*^2^ = 0.008].

### Relationships Between Prosocial Behavior and Social-Emotional and Behavioral Competence

Results of correlational analyses carried out on the entire group of participants to assess associations between prosocial behavior measures and social-emotional and behavioral variables, controlling for children’s age, are shown in Table 3.

**Table 3:** Pearson partial correlations performed between prosocial and social-emotional and behavioral measures (values of *p* in brackets).

	Anxiety-withdrawal score	Anger-aggression score	Social competence score	TEC score
Total prosocial behaviors	−0.024 (0.765)	−0.134 (0.101)	0.371(<0.001)	0.150 (0.066)
Spontaneous prosocial behaviors	−0.178 (0.029)	0.122 (0.135)	0.080 (0.328)	0.048 (0.555)
Requested prosocial behaviors	−0.045 (0.584)	−0.159 (0.052)	0.126 (0.124)	0.164 (0.044)

Children who were rated by their teachers as more socially competent were also those who exhibited more prosocial behaviors during spontaneous interactions with their peers; moreover, the same children also showed a more advanced level of emotion understanding, although the correlation calculated was only marginally statistically significant. In addition, emotion comprehension was also positively associated with the proportion of prosocial behaviors enacted following an explicit or implicit request by a peer; this last variable is marginally negatively associated with the anger-aggression dimension scores. Finally, in children who were described as having higher levels of anxiety and withdrawal a fewer spontaneous prosocial acts were observed too.

## Discussion

The main goal of this study was to investigate whether and how early group experience, such as that of children attending daycare centers, may or not influence the predisposition to enact prosocial behaviors throughout the preschool years. To summarize the results, no differences were found between the two groups of children examined concerning the productivity of prosocial behaviors; however, some differences emerged in the quality of enacted behaviors that appeared to be associated with certain social-emotional and behavioral characteristics.

Indeed, the daycare experience requires children to create new relationships outside the family very early on, and this means to engage in interactions with peers, to adapt to teachers’ expectations and demands, and to test and modify their social-emotional abilities by experience (Hyson and Taylor, 2011; Grazzani et al., 2016). As some studies have shown, while attending daycare children are frequently exposed to prosocial behaviors and, consequently, they are prone to enact such behaviors themselves for two main reasons: one refers to social imitation processing, the other to the evidence that through the care that the young children receive, they learn to care for others (Quigley and Hall, 2016; McCormick, 2018; Bleiker et al., 2019; Schmerse and Hepach, 2021). Furthermore, the study of Over and Carpenter (2009) evidenced that the mere condition of familiarizing children with photographs in which group situations are represented, and thus clearly affiliative and social, leads them to enact more helping behaviors than children who have been exposed to non-affiliative pictures, in which isolated individuals are represented. Therefore, it is legitimate to hypothesize that this early group experience may encourage the development of positive social behaviors.

However, previous studies found discrepant results in this regard because children who attended daycare are generally considered more socially competent, but it is not so clear whether they are also more prosocial than those who did not attend it (Erel et al., 2000; Belsky et al., 2007; Ansari, 2018; Bleiker et al., 2019). At the same time, daycare attendees may also be more involved in and exposed to aggressive behaviors among peers, as children exhibit their peak of physical aggression generally between 18 and 30 months of age (Huston et al., 2015; Pingault et al., 2015; Filho et al., 2016; Ansari, 2018). Observing other children’s aggressive behavior has a noticeable effect, as children tend to imitate it. In this regard, as reported by several authors, it is important to specify that aggressive attitudes can themselves be considered adaptive; in fact, in some circumstances, it is feasible that assertiveness may be confused with aggression or disobedience, especially in the case of toddlers and preschoolers who are not yet sufficiently competent to negotiate in a mature assertive way with others (NICHD Early Child Care Research Network, 1998, 2001, 2003; Pingault et al., 2015; Ansari, 2018).

The results of this study revealed no noteworthy differences in the overall production of children’s prosocial behaviors enacted during a free play situation attributable to their previous daycare experience. Similarly, no differences in teachers’ evaluations of the social competence level of the two groups of children were found, as measured by the corresponding subscale of the SCBE-30. It is relevant to specify that the Social Competence subscale of SCBE-30 mainly refers to prosocial behaviors; thus, this result may represent further confirmation of the similarity in the level of prosocial skills that characterized the two groups of children considered. Moreover, the positive association found, in the whole sample, between the scores on this SCBE-30 subscale and the total number of prosocial acts detected by direct observation further confirm that the two measures considered refer to the same construct.

However, differences emerged between the two groups concerning the origin of the observed prosocial behaviors, self-initiated or requested; specifically, children who have not attended daycare engaged in prosocial acts required by peers more than those who had a previous daycare experience. To interpret this result, it may be helpful to consider the social-emotional and behavioral characteristics of these children who, in the judgment of teachers, were more anxious, solitary, and withdrawn (SCBE-30 Anxiety-Withdrawal subscale score). For this reason, they may be more susceptible to external solicitations and act prosocially when prompted. On the other hand, the findings obtained also indicated that children with previous daycare experience were rated by their teachers as having more externalizing behaviors (SCBE Anger-Aggression subscale score). Other studies found similar findings, emphasizing that these children were more likely to exhibit aggressive behaviors and engage in conflict with other children, not only at preschool age but also later in life.

In this study, these characteristics appeared to negatively influence the propensity to respond to others’ requests for positive social behaviors, as evidenced by the correlation, on the whole sample, between the SCBE Anger-Aggression subscale score and the proportion of requested prosocial acts.

Such a result can be interpreted considering those previous studies that have highlighted how negative emotionality (anxiety, sadness, fear, and rage) can inhibit prosocial behavior (Taylor et al., 2014; Edwards et al., 2015; Xiao et al., 2019), although they had not distinguished between spontaneous and required acts. This view was also supported by the negative association between anxiety-withdrawal scores and the propensity to spontaneously produce prosocial acts, found when considering all participants in this study. Overall, the main social-emotional and behavioral differences found between groups seemed to influence more the propensity to engage in prosocial acts on request than spontaneously.

Concerning the motives underlying prosocial behavior, children who had previously attended daycare showed, proportionately, more behaviors directed toward satisfying the desire of another person. So, an early group experience could support the ability to correctly identify and interpret the intentions of others and respond to them appropriately, enacting the right helping action (Newton et al., 2014). However, daycare experience seemed to have an opposite effect on prosocial actions supported by affiliative motivations, which were lower in children who have attended daycare. This outcome appears congruent with the fact that children who had experienced various forms of non-maternal care during the first years of life were described as less compliant with adults and more transgressive (NICHD Early Child Care Research Network, 1998, 2001, 2003, 2006; Varin, 2007) so they may be less likely to engage in prosocial behavior based on shared social rules.

In addition, the main differences between the two groups of children cannot be explained by emotion comprehension since they did not differ in this ability. According to this outcome, the two groups were also similar in the rate of prosocial acts driven by the emphatic comprehension of others’ needs. However, in line with previous literature (Rose-Krasnor and Denham, 2011; Grazzani et al., 2016), emotion comprehension ability was positively associated with the propension to engage in prosocial behaviors, particularly those on request, as we found in the whole sample. In this regard, it is important to remember that, according to our coding system, also implicit requests of emotion regulation were considered; therefore, this ability might play a principal role when requests for help are implicit, that is when they are expressed indirectly by specific signals of a negative emotional state. These signals are species-specific, and children with a greater ability to understand emotions might detect them better (Tomasello, 2019).

On the whole, the present study has shown that the differences in prosocial behavior of children who have attended daycare or who have not can be attributed, at least partially, to some social-emotional and behavioral characteristics that distinguish them. Among these, the propensity to anger-aggression, on the one hand, and anxiety-withdrawal, on the other, were those that seemed to affect more the quality of prosocial behaviors, rather than their quantity. In other words, this study suggested that the incidental effects of daycare on prosocial behavior might be canceled due to the peculiar social-emotional and behavioral characteristics of the two groups of children.

The use of naturalistic observation as a method of measurement permitted to distinguish between spontaneous and requested prosocial acts, allowing to obtain new suggestions about the controversial issue concerning the links between prosocial behavior and children’s social-emotional characteristics. As anticipated, most previous studies have been conducted by parent and/or teacher evaluations and experimental designs. In this respect, it is worth noting that adult evaluations might be influenced by biases (Bouchard et al., 2020), whereas in experimental designs, children might be influenced by social expectations; in fact, at approximately 5 years of age, children come to be concerned about their reputations and show the emergence of self-promotional strategies, increasing prosociality in public compared to private settings (Engelmann and Rapp, 2018; Rapp et al., 2019). Thus, naturalistic observations of children’s behaviors allow to overcome these potential limitations and obtain some precise information about the antecedents and recipients of children’s prosocial acts.

However, this study presents some limitations concerning relevant information about the early caring experiences of the two groups examined. In particular, the main lack of information about children with daycare experience concerned both the age of entry and the average daily time spent in that context, and its quality. Instead, for the other group of children, we have no data available regarding some features such as primary caregivers, caring strategies, and frequency of peer relationships.

All of these variables can influence children’s social-emotional development, accounting for different patterns of outcomes that can be observed in children who have attended daycare, as shown by several studies (Varin, 2007; Pingault et al., 2015; Bulgarelli and Molina, 2016). Similarly, family socioeconomic status was not directly considered in this work. However, there is some evidence that the main differences between children with and without daycare experience were modulated by this variable since the positive effects of daycare attendance appeared to be greater for children with a low SES (Andersson, 1992; Ansari, 2018).

In addition, given the wide variability associated with the age of the participants, their preschool attendance also differed; this may have impacted, at least in part, the effects attributable to the previous daycare experience, although the age variable was controlled for in the analyses performed.

Moreover, important developmental changes in various social-cognitive skills occur during the preschool period, including theory of mind (Peterson and Wellman, 2019), self-regulation (Montroy et al., 2016), emotion understanding (Pons and Harris, 2005). Consequently, children’s social behavior may result from different mechanisms depending on the specific age under consideration (Eisenberg et al., 2011). For these reasons, it would be appropriate to conduct longitudinal studies in order to better define the effect of daycare attendance over time.

## Conclusion

The current study represents one of the first attempts to examine the long-term effects of daycare attendance on preschoolers’ prosocial behaviors by using direct observation of their spontaneous interactive exchanges. This choice allowed to generalize some results already present in the literature and mainly focused on prompted prosocial behavior in structured conditions, extending them to the natural context of kindergarten.

New data are provided confirming that daycare attendance does not appear to have an impact on the enactment of prosocial behaviors when considering the total amount of such behaviors, not only in the immediate (Bleiker et al., 2019), but also in later periods (Pingault et al., 2015; Schmerse and Hepach, 2021). However, at the same time, this early socialization experience appears to negatively influence, specifically, the production of prosocial acts following a request from a peer. This evidence suggests the need to consider the possible role that the social partner (an adult rather than another child) may assume in encouraging or not the occurrence of prosocial acts. In addition, as supported by other studies (Schuhmacher et al., 2017), some social factors, including daycare attendance, as well as individual factors, such as social-emotional and behavioral abilities, may differentially influence the motivations underlying prosocial action, accounting for the distinctiveness of different types of prosocial behavior observed.

## Data Availability Statement

The original contributions presented in the study are included in the article and further inquiries can be directed to the corresponding author.

## Ethics Statement

The studies involving human participants were reviewed and approved by Research Ethical Committee of the University of Milano-Bicocca. Written informed consent to participate in this study was provided by the participants’ legal guardian/next of kin.

## Author Contributions

NS and CC: conceptualization and methodology, data collection, data coding and analysis, writing original draft, review and editing. All authors contributed to the article and approved the submitted version.

## Conflict of Interest

The authors declare that the research was conducted in the absence of any commercial or financial relationships that could be construed as a potential conflict of interest.

## Publisher’s Note

All claims expressed in this article are solely those of the authors and do not necessarily represent those of their affiliated organizations, or those of the publisher, the editors and the reviewers. Any product that may be evaluated in this article, or claim that may be made by its manufacturer, is not guaranteed or endorsed by the publisher.
